# Establishment of fishing cat cell biobanking for sustainable conservation

**DOI:** 10.3389/fvets.2022.989670

**Published:** 2022-11-10

**Authors:** Woranop Sukparangsi, Ampika Thongphakdee, Santhita Karoon, Nattakorn Suban Na Ayuthaya, Intira Hengkhunthod, Ratchapon Prakongkaew, Rungnapa Bootsri, Wiewaree Sikaeo

**Affiliations:** ^1^Department of Biology, Faculty of Science, Burapha University, Chon Buri, Thailand; ^2^Wildlife Reproductive Innovation Center, Animal Conservation and Research Institute, Zoological Park Organization of Thailand Under the Royal Patronage of H.M. the King, Bangkok, Thailand

**Keywords:** cryopreservation, biobank, fishing cat (*Prionailurus viverrinus*), wild felid, fibroblast, reprogramming

## Abstract

The fishing cat (*Prionailurus viverrinus*) is a vulnerable wild felid that is currently under threat from habitat destruction and other human activities. The zoo provides insurance to ensure the survival of the fishing cat population. Creating a biobank of fishing cats is a critical component of recent zoo strategies for securely stocking cell samples for long-term survival. Here, our goal was to compare cell biobanking techniques (tissue collection, primary culture, and reprogramming) and tissue sources (ear skin, abdominal skin, testis) from captive (*n* = 6)/natural (*n* = 6) vs. living (*n* = 8)/postmortem (*n* = 4) fishing cats. First, we show that dermal fibroblasts from the medial border of the helix of the ear pinna and abdominal tissues of living fishing cats can be obtained, whereas postmortem animals provided far fewer fibroblasts from the ears than from the testes. Furthermore, we can extract putative adult spermatogonial stem cells from the postmortem fishing cat's testes. The main barrier to expanding adult fibroblasts was early senescence, which can be overcome by overexpressing reprogramming factors through felid-specific transfection programs, though we demonstrated that reaching iPSC state from adult fibroblasts of fishing cats was ineffective with current virus-free mammal-based induction approaches. Taken together, the success of isolating and expanding primary cells is dependent on a number of factors, including tissue sources, tissue handling, and nature of limited replicative lifespan of the adult fibroblasts. This study provides recommendations for tissue collection and culture procedures for zoological research to facilitate the preservation of cells from both postmortem and living felids.

## Introduction

Fishing cat (*Prionailurus viverrinus*) is one of the small-medium wild cat species distributed in Thailand (1–4). Fishing cats have an essential role in the wetland ecosystem; however, in recent years, their wetland habitats in Thailand and Southeast Asia have been destroyed by human threats, such as the prawn farming industry, illegally established aquaculture ponds, human settlement, excessive hunting, deforestation, depletion of fish stocks from overfishing, and incidental poisoning (5–8). The fishing cat is now included in the “vulnerable” status (IUCN Red List of Threatened Species) (9) and CITES Appendix II and is protected by national legislation. The captive fishing cats in the Zoological Park Organization of Thailand are currently under conservation programs aimed at improving their breeding and producing more offspring, which can eventually be reintroduced back to natural habitats. Rescuing endangered or vulnerable wildlife animals through the zoo breeding program involves assisted reproductive technology (ART), which has become an urgent trend to secure valuable genetics for long-term conservation. The storage of valuable genetics from wild animals can be performed in several ways, including cryopreservation of gametes (spermatozoa and oocyte) and embryos for future *in vitro* fertilization (IVF), artificial insemination (AI), and embryo transfer (10–12). To date, Santymire et al. (13) and Thongphakdee et al. (14) provided a better understanding of reproductive biology in fishing cats and demonstrated a potential application in their captive breeding. However, there are some restrictions to developing ART in fishing cats, including a restricted number of fertile fishing cats, high variability in the ovarian response to estrus and ovulation induction for AI (14), and low success in fertilization (15).

Another approach to secure good genetics of fishing cats as well as other wild animals is to establish primary cells, in particular fibroblasts with some restricted degree of propagation and stem cell lines such as embryonic stem cells (ESCs), mesenchymal stem cells (MSCs), and induced pluripotent stem cells (iPSCs) with more capacity to self-renewal and differentiation. The preservation of these living samples as well as gametes and embryos in a liquid nitrogen-containing cryotank generates a viable cell bank repository of wildlife (as demonstrated by the San Diego Zoo's “Frozen Zoo^®^”) for future cell-based conservation approaches (16, 17). This includes somatic cell nuclear transfer (SCNT) or animal cloning to produce new offspring and developing stem cell-based generation of synthetic embryo blastocysts [(18) and reviewed in Shahbazi et al. (19)], which requires further studies in Felidae. *In vitro* culture of various cell types also provides a great benefit of reducing and replacing using animals for experimentation in the 3R model, namely, the reduction, refinement, and replacement model (20).

Primary culture tissue can be harvested from live animals or freshly dead/postmortem animals (21–23). Fibroblast culture has already been achieved in numerous species of mammals, mainly in mice (*Mus musculus*) and humans and in other mammals, such as horses, dogs, drills, rhinoceros, and elephants (23–26). In felid species, fibroblasts were derived in domestic cats for the generation of iPSC and intraspecific feeder cells to support the induction of the derivation of iPSC and cat ESC and in wild cats, including Bengal tiger, jaguar, serval, and snow leopard (27–30). Therefore, in this study, our objective is to establish cell biobanking for fishing cats to preserve their genetics for long-term conservation. During a decade of fishing cat projects in our zoo organization, we report successful attempts to derive fibroblasts from different sources, including living and dead fishing cats in captivity and natural resources. Here, we also examine the usability of our fibroblasts for cellular reprogramming and provide information on the current challenges to these approaches.

## Methods

### Animal ethics

This study was approved under the project “Development of fundamental science and innovation for sustainable fishing cat conservation” with subproject “Conservation of fishing cats (*Prionailurus viverrinus*) by using the innovation of stem cell technology innovation” by the Wild Animal Ethics Committee of Zoological Park Organization of Thailand under the Royal Patronage of H.M. the King (Protocol number: 630960000030 granted to PI of the project—A.T.). The Department of Wildlife and Plant Conservation, Thailand, granted permission to work with wild fishermen in the natural parks of Thailand [Permission number: Tor Sor (in Thai) 0907.4/17939 (issued date: 22/09/2021)]. The collection of tissue from postmortem fishing cats and during artificial insemination was conducted by veterinarians at the Animal Hospital Unit, Khao Kheow Open Zoo (KKOZ), Zoological Park Organization, Chon Buri, Thailand. Cell culture was carried out at the Wildlife Reproductive Innovation Center (WRIC), Research Department, Bureau of Conservation and Research, KKOZ. The genetic materials and chemicals in this study were used under the Biosafety Certificate (22/2559) approved by the Biosafety Committee of Burapha University.

### Primary culture and cryopreservation

The primary culture with tissue explant method was adapted from Gómez et al. (31). Briefly, the collected tissues (skin and testis) were washed with Dulbecco phosphate-buffered saline (DPBS, ThermoFisher) containing 500 μL (in 50 mL DPBS) of penicillin (10,000 unit/mL)-streptomycin (10 mg/mL) (PS) solution (Sartorius) and 500 μL (in 50 mL DPBS) of 10X Amphotericin B (AmB) solution (Sartorius) for 3 times. Removing intact subcutaneous adipose tissues and hair (for skin tissues) was conducted to avoid bacterial and fungal contamination and the presence of lipid droplets. To activate the fibroblast outgrowth of tissues using the wound healing process, several small cuts were made using a scalpel and the tissues were cut in ~2 × 2 mm. The excised tissues were then placed (3–4 pieces) in a gelatin-coated plate (Attachment Factor, ThermoFisher)-coated dish (35 mm) or 12-well plates for 10 min for better explant attachment. Then, a warm complete fibroblast medium was added to the dish/plate with the lowest volume as possible to avoid floating tissue and ensure outgrowth. The primary culture was placed in the CO_2_ incubator (5% CO_2_ in humidified 95% air at 38.0°C). To avoid contamination, the cultures were monitored every day, and the medium changes were performed every day without disturbing the explant attachment. Complete fibroblast medium (50 mL) consisted of 43 mL of Dulbecco's Modified Eagle Medium (DMEM) high-glucose medium (ThermoFisher), 5 mL (10% in all experiments, unless stated otherwise) and 10 mL (20% used only in the medium test) of fetal bovine serum (FBS) (Sartorius), 500 μL of MEM non-essential amino acid solution (NEAA, Sigma, Merck), 500 μL of Glutamax (ThermoFisher), 500 μL of sodium pyruvate (Sigma, Merck), and 500 μL of antibiotics PS and 500 μL of antimycotics AmB (Sartorius). To test medium conditions for fibroblast growth support, each supplement was replaced with an equal amount of DMEM. For cryopreservation, cells were resuspended in a freezing medium containing a complete fibroblast medium with 10% dimethyl sulfoxide (DMSO) or Recovery Cell Culture Freezing Medium (ThermoFisher) and placed at −80°C overnight using Mr. Frosty^TM^ Freezing Container and the next day transferred to liquid nitrogen for long-term storage. All cells in this study are currently stored in the Liquid Nitrogen Tank Facility of the Thailand Biobank under the Zoological Park Organization (ZPO). Further details of cell storage and material transfer are available upon request from the corresponding author or the ZPO Research Bureau.

### MTS assay

The MTS colorimetric assay kit (Abcam), described as a one-step MTT assay, was used to detect mitochondrial activity to indirectly quantify the cell viability (the reduction of the MTS tetrazolium by viable cells generates a colored and soluble formazan product) (32). MTS was chosen for this study because it produced a less toxic formazan product, and the cells can be returned to cell culture and evaluated further (32). For nucleofection, transfected cells were seeded in a 96-well plate (10,000 cells/well) in the presence or absence of the RevitaCell^TM^ supplement (Gibco). The RevitaCell^TM^ supplement was added at the final concentration of 0.5X. On day 4 after nucleofection, the fresh fibroblast medium was changed before performing the MTS assay (200 μL/well). Then, 20 μL of MTS solution was added and incubated for 4 h at 37°C. Absorbance at 490 nm was measured by a microplate reader (Thermo Scientific^TM^ Multiskan^TM^ GO Microplate Spectrophotometer) with Skanlt^TM^ software. Four independent experiments with three technical replications each were performed to measure cell viability.

### Immunofluorescence

To observe the presence of spermatogonial stem cells (SSCs), seminiferous tubule extracts of the fishing cat in 24-well plates were fixed with 500 μL of 4% paraformaldehyde (PFA) for 15 min at room temperature and washed with 500 μL of DPBS 3 times. To permeabilize the plasma membrane, fixed cells were treated with 500 μL of 0.1% Triton X-100 in PBS for 15 min at room temperature and washed with 500 μL of DPBS 3 times and each time incubated for 5 min. The cells were then treated with 500 μL of 1% BSA in DPBS for 1 h at room temperature. Cells were treated with primary antibodies SOX2 (AB5603, Merck), at dilution of 1:300 in 500 μL of 1% BSA in DPBS, overnight at 4°C and washed with 500 μL of DPBS 3 times the next day before secondary antibody staining. Cells were stained with Alexa Fluor 647 (ThermoFisher, 1:800) and Hoechst33342 (ThermoFisher, 1 μg/mL) in 500 μL of 1% BSA in DPBS in the dark for 1 h at room temperature and washed with 500 μL of DPBS 3 times. Fluorescent micrographs were taken with an Eclipse Ti-S Inverted Research Microscope (Nikon) and a digital camera.

### Nucleofection

Nucleofection^TM^ programs in the 4D-Nucleofector^TM^ system included CA-137 (specific for human iPSC and mammalian fibroblasts with primary cell 2 (P2) and 3 (P3) solution kit), DS-150, EH-100, EN-150, EO-114 (specific for mammalian fibroblast recommended by Lonza and compatible with the P2 solution kit), FF-135 [specific for human fibroblast and dental palp cells (33–35)], and DT-130 [specific for normal newborn human dermal fibroblast, NHDF-neo cell lines compatible with P2 solution and human fibroblast (36)]. Small-scale 4D-Nucleofector^TM^ kits [P2 Primary Cell 4D-Nucleofector^®^ X Kit S 32 RCT, V4XP-2032), composed of a 16-well Nucleocuvette^TM^ strip and a primary cell 2 (P2) solution, were used for nucleofection. To prepare for small-scale fibroblast cell transfection, the fibroblast cells at P.1 (70% confluency in a 35-mm dish) were subcultured, counted by hemocytometer, transferred 10^5^ cells to a new microcentrifuge tube, and then centrifuged at 90 g for 5 min, the supernatant was aspirated supernatant, and the cell pellet was gently resuspended in 20 μL of P2 solution; 400 ng of pmaxGFP^TM^ vector was added and gently mixed, transferred cell suspension to Nucleocuvette^TM^ Strip, nucleofected with programs of interest, and kept the transfected cell suspension at room temperature for 10 min; 80 μL of complete fibroblast medium (CF) was added to Nucleocuvette^TM^, transferred transfected cells into noncoated 4-well plates containing 420 μL complete fibroblast medium per well and incubating cells in a CO_2_ incubator at 38°C. GFP expression was observed under a fluorescence microscope.

### Senescence test

Senescence β-galactosidase staining (Abcam) was used to detect the sign of cell senescence according to the manufacturer's instructions. Briefly, for 6-well plates, the fibroblasts were washed once with 1 mL of DPBS, fixed with 1 mL of fixative solution for 10 min, and then washed twice with 1 mL of DPBS. The cells were then stained with 1 mL of a staining solution mix containing 930 μL of 1× staining solution, 10 μL of 100X Solution A, 10 μL of 100X Solution B, and 50 μL of 20 mg/mL X-gal and incubated overnight at 37°C. The next day, stained cells were stored in 70% glycerol before imaging with an inverted microscope. To interpret the result of senescence test, more intensity of blue color in cells reflects more senescence.

### Flow cytometry analysis

To monitor transfection efficiency, GFP expression was detected by flow cytometry (FlowSight^®^ Imaging Flow Cytometer, Luminex). On day 4 after nucleofection, the transfected cells were washed once with DPBS, dissociated with 0.25% Trypsin-EDTA for 3 min, neutralized with fibroblast medium, split down the cell pellet, and resuspended in DPBS. Hoechst33342 (1 μg/mL) was then added to the cell suspension to stain all cells. Flow cytometric data were analyzed using FCS Express 7 software. The GFP/Hoechst33342 cell populations were gated, and transfection efficiency was calculated from the percentage of double-positive cell populations with GFP and Hoechst33342 vs. the total Hoechst33342-positive cells. Four independent experiments were performed to measure transfection efficiency.

### Reprogramming assay

In all experiments, only fibroblasts at P.1–2 were used for reprogramming. The PiggyBAC transposon (PB) vector MKOS-mOrange (gifted by Dr. Keisuke Kaji) contains 4 mouse reprogramming factors: C-Myc, Klf4, Oct4, and Sox2 under CAG promoter and mOrange reporter. The DNA vector was transfected by nucleofection. Abdominal fibroblasts were used as somatic cells for this mouse-based PiggyBAC system. The transfected cells were cultured with iPSC induction medium containing advanced DMEM (ThermoFisher), 10% FBS, MEM non-essential amino acid solution (NEAA, Sigma, Merck), Glutamax (ThermoFisher), sodium pyruvate (Sigma, Merck), and antibiotics PS-antimycotics AmB (Sartorius) with 10 ng/mL of human leukemia inhibitory factor (hLIF, Peprotech), unless otherwise stated. The cells were reseeded onto irradiated MEFs or other coating matrices, including attachment factor/gelatin (ThermoFisher), Geltrex (ThermoFisher), and vitronectin (ThermoFisher). The second PB method used PB vector called pC6F (addgene: 140826), containing a Tet-On system that regulates the expression of polycistronic cassettes of six reprogramming factors: human OCT4, SOX2, KLF4, C-MYC, KLF2, and NANOG and reporter tdTomato (37). We transfected pC6F in conjunction with the transposase vector (pCy43, Sanger Institute) and the PB rtTA vector (addgene: 126034) using Lipofectamine 3000 (ThermoFisher) overnight in the testicular fibroblasts. Transfection was carried out once, and on day 3, we selected cells carrying the pC6F construct with puromycin (2 μg/mL) and induced the expression of reprogramming factors with doxycycline in the induction medium of iPSC.

The episomal vector system is composed of pCXLE-hSK (vector with SOX2 and KLF4; addgene: 27078), pCXLE-hUL (vector with L-MYC and LIN28; addgene: 27080), pCXLE-hOCT3/4 (vector with OCT3/4 (POU5F1); addgene: 27076), pCXWB-EBNA1 (vector with EBNA1; addgene: 37624). These vectors were transfected into the abdominal fibroblasts using Nucleofector. The transfected cells were cultured in iPSC medium, unless otherwise indicated. Commercial media were also tested for reprogramming, including NutriStem hPSC XF medium (Sartorius), medium containing Knockout Serum Replacement (KOSR, ThermoFisher), and Essential 8 medium (ThermoFisher).

For the self-replicating RNA (srRNA) system, we generated RNA from the plasmids T7-VEE-OKSiM, T7-VEE-OKSiG, and T7-VEE-GFP plasmids using the *in vitro* transcription technique with HiScribe^TM^ T7 quick high-yield RNA synthesis kit, according to the manufacturer's protocol. Synthesized RNA was modified by the Vaccinia Capping System (NEB), the mRNA cap 2′-O-methyltransferase (NEB), and *E*. *coli* poly (A) polymerase (NEB). The abdominal fibroblasts were transfected with srRNA using Lipofectamine^TM^ MessengerMax^TM^ (ThermoFisher) according to the Yoshioka and Dowdy modified method. In brief, the abdominal fibroblasts were transfected with self-replicating RNA (625 ng/well) for 3 h in the presence of 200 ng/mL of recombinant viral B18R protein (R&D Systems). The transfection medium was changed to complete fibroblast medium after incubation. The transfected cells were cultured in an iPSC medium with 200 ng/mL of B18R. B18R was removed once the transfected cells were ready to be seeded in the irradiated MEF. PiggyBAC MKOS-mOrange (female fishing cat#4, Table 1), PiggyBAC pC6F (male fishing cat#5, Table 1), and episomal reprogramming experiments (female fishing cat#4, Table 1) were carried out in 6-well plates with starting cells at 10^5^ cells/well. Self-replicating RNA reprogramming experiments (female fishing cat#4, Table 1) were carried out in 24-well plates with 5 × 10^4^ cells/well.

**Table 1:** Primary culture of fishing cat tissues from various sources.

**Sources for tissue collection**	**Sex/status during tissue collection** ** [living (L); postmortem (P)]**	**Age (y, year; m, month) at time of tissue collection**	**Transportation time until explant seeding/medium** ** (all stored in 4°C)**	**Outgrowth (days)**	**#Re-explant** ** (round)**	**#Cryovials (each vial contains ~10^5^ cells)/number of cell line**
**Natural resources**						
Ear pinna	Male#1/L	Subadult 1y	5 h/FB	3	3	19/2
Ear pinna	Female#1/L	Subadult 1y	5 h/FB	3	3	11/2
Ear pinna	Female#2/L	Subadult 1y 2m	3 days/Saline	0	0	0
Ear pinna	Male#2/L	Adult 2y 8m	1 day/FB	0	0	0
Ear pinna	Male#3/L	Subadult 1y	1 day/FB	0	0	0
Ear pinna	Female#3/L	Adult >2.5y	2 days/FB	0	0	0
**Captivity**						
Abdomen	Female#4/L	Adult 13y	1 h/DMEM	15	0	7/1
Abdomen	Female#5/L	Adult 12y	1 h/DMEM	13	0	3/1
Ear pinna	Female#6/P	Adult 16y	1 h/FB	13	0	0
Ear pinna	Female#7/P	Baby 2m	1 h/FB	5	0	2
Ear pinna	Male#5/P	Adult 12y	5 h/FB	0	0	0
Testis	Male#5/P	Adult 12y	5 h/FB	3	2	5/1

### Alkaline phosphatase staining of live cells

Fluorescein-based live alkaline phosphatase (Live-AP) stain (ThermoFisher) was used to detect AP activity in cell extracts from seminiferous tubules and epididymis. In brief, we prepared a diluted AP stain (final concentration: 1:500) and nuclear staining solution Hoechst33342 (final concentration: 10 μg/mL) mixed with advanced DMEM medium (2 μL of Live-AP in 1 mL medium in 6-well plates) and stained cells for 20 min before imaging with a fluorescent microscope.

### Statistical analysis

Data from three to four independent experiments (with at least three technical replicates) are presented as the mean ± standard deviation (SD). Statistical analyses were performed using Student's *t*-test to determine statistical significance between the groups.

## Results

### Culture of adult dermal fibroblasts from the abdominal dermis of living fishing cats

**Figure 1:**
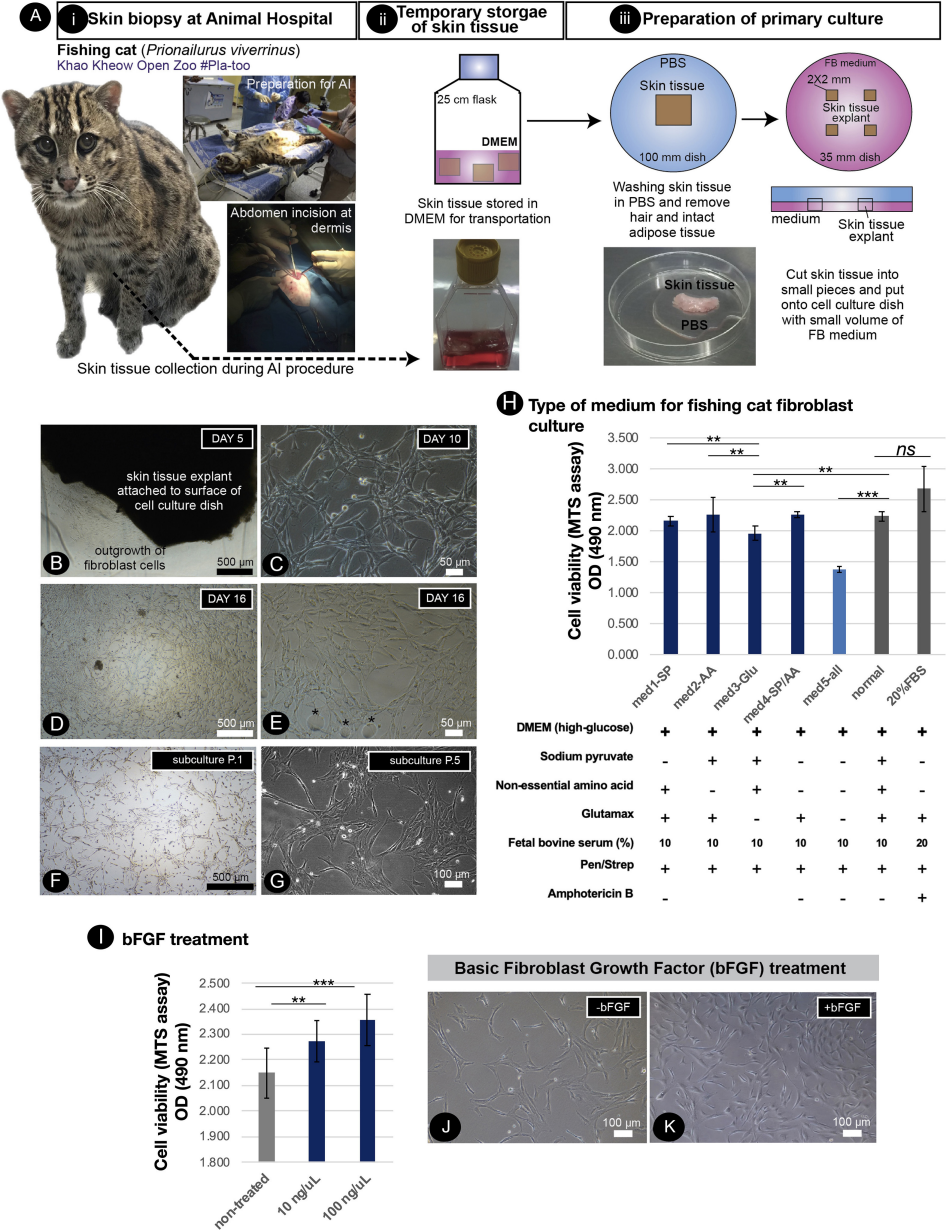
Primary culture of fishing cat cells and conditions supporting fishing cat cell expansion. **(A)** Tissue collection and preparation for primary culture: (i) skin biopsy was performed by veterinarians during artificial insemination of fishing cat. (ii) The skin tissues were then transported to the Tissue Culture Facility in DMEM or complete fibroblast (FB) medium supplemented with antibiotics. (iii) The skin tissues were washed with PBS and dissected into small pieces, and the tissue explants were cultured in FB medium. **(B)** The adult fibroblasts were expanded from skin tissue explant as early as day 5. **(C)** Primary culture shows the morphology of fishing cat fibroblast at day 10. **(D)** Fibroblasts expanded and reached 80% confluency within 16 days. **(E)** Primary culture contained some oil droplets (*) due to high subcutaneous fat connected to the dermis layer. **(F,G)** Culture of the adult dermal fibroblasts after subculture from the primary culture. **(H)** Adult dermal fibroblasts from the fishing cat at passages 2–4 were cultured in different medium conditions. Bar graph shows cell viability of fibroblasts under different medium conditions at day 7 post-treatment. The cell viability was measured by the MTS assay by spectrophotometry (at 490 nm). **(I)** The fibroblasts were treated with basic fibroblast growth factor (bFGF) at final concentrations 10 and 100 ng/μL. The bar graph shows MTS assay-based cell viability of fibroblasts at day 4 post-bFGF treatment. **(J)** Brightfield photograph showing the morphology of fibroblasts without bFGF. **(K)** Brightfield photograph shows morphology of fibroblasts with bFGF at day 2 post-treatment. Asterisks (** and ***) indicate significant differences (*p* < 0.05 and *p* < 0.01, respectively, Student's *t*-test), and “ns” indicates not significant differences (*p* < 0.05).

Skin biopsy to collect the dermis to produce fibroblast culture, an available source of cellular reprogramming, from living fishing cats, is limited due to the invasive procedures required. Within the assisted reproductive technology (ART) program of the zoo, direct extraction of the dermis on the abdominal skin of a fishing cat was conducted during the artificial insemination (AI) operation (Figure 1Ai–iii), allowing us to derive dermal fibroblasts from a female fishing cat. As shown in Figure 1B, the fibroblast outgrowth appeared around the edge of the tissue explants in 5 days. No epithelial cells were detected, indicating the absence of keratinocytes (Figures 1B–E). The dermal fibroblasts in the primary culture expanded to reach their 90% confluency on day 16 with a homogeneous fibroblastic morphology (Figure 1D). However, since the collection of tissue from the abdominal dermis was closed to the subcutaneous layer that contained rich adipose tissue, lipid droplets were found during culture (Figure 1E). A secondary culture was achieved by removing explant tissues and subcultured the fibroblasts into appropriate culture vessels (split ratio of 1:2; cell density: 2.0 × 10^4^ cells/cm^2^). The fibroblasts expanded to reach their 90% confluence within 4 days after first passaging (Figure 1F). It should be noted that seeding the fishing cat fibroblasts at a too low cell density led to less cell expansion. In the early passages, the fibroblasts exhibited a spindle shape (Figure 1F) while at later time the cell area expanded in a less nucleus:cytoplasm ratio (Figure 1G). In the later passages (P.4–5), the fibroblast culture was affected by the longer trypsinization time required for subculture and less cell expansion. In addition, by removing supplements and measuring the cell division rate with the MTS assay, we also show the essential components of fibroblast medium to support cell growth and division. As shown in Figure 1H, the removal of sodium pyruvate or non-essential amino acids or both did not reduce cell proliferation while the removal of Glutamax caused a significant reduction in cell proliferation. The effect became prominent with the complete removal of all supplements. Thus, additional supplements helped to improve the expansion of fibroblast cells. Furthermore, increasing the percentage of fetal bovine serum (FBS) from 10 to 20% did not significantly improve cell proliferation (Figure 1H). To improve fibroblast culture, we next tested the addition of basic fibroblast growth factor (bFGF or FGF2) and found that bFGF improved fibroblast proliferation (Figures 1I–K).

### Adult dermal fibroblast culture from the pinna of the ears of living fishing cats

**Figure 2:**
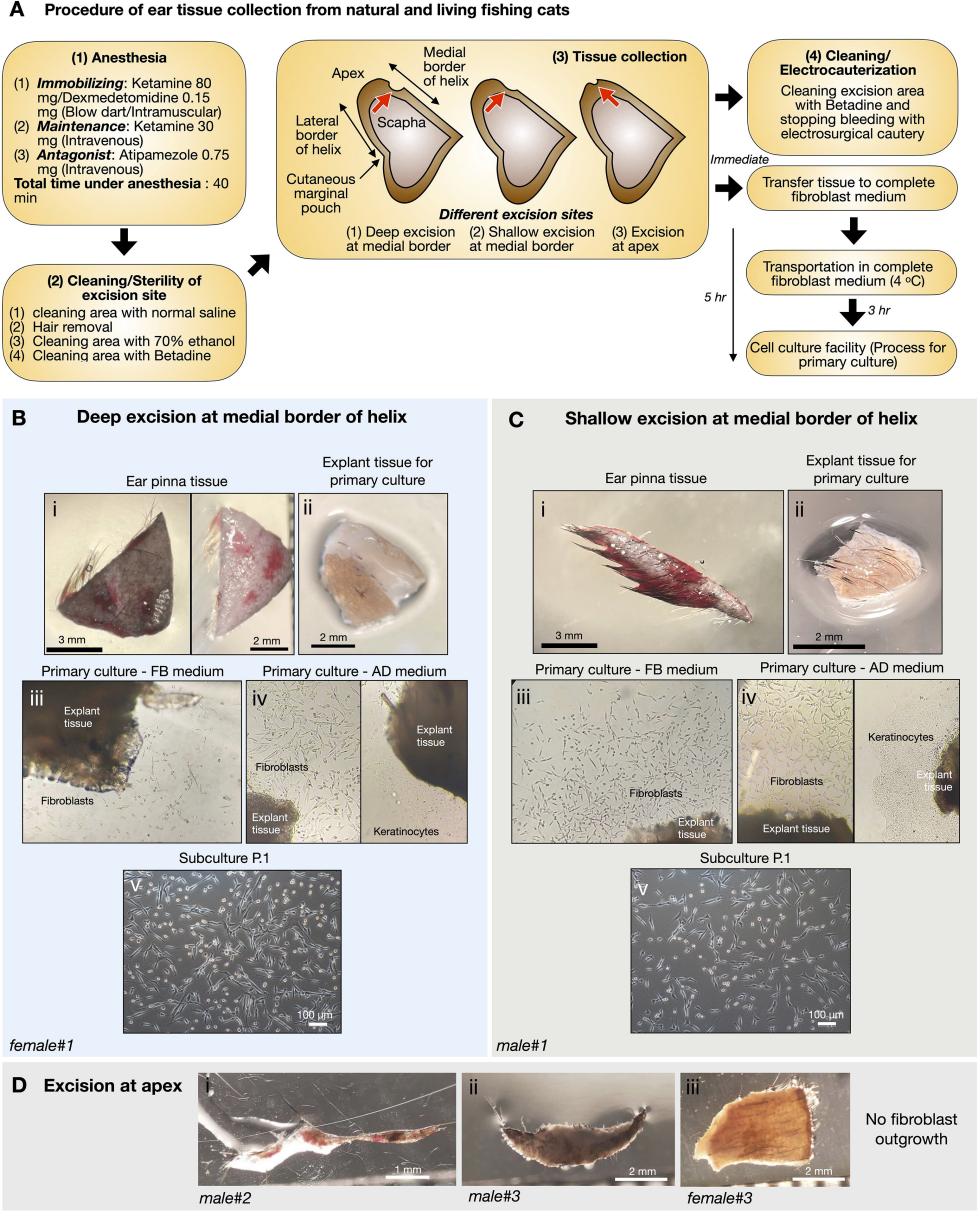
Primary culture of fishing cat tissues collected from natural resources. **(A)** Procedure of ear tissue collection from natural and living fishing cats. **(B,C)** Tissues from excision at medial border of helix of ear pinna from female fishing cat **(B)** and male fishing cat **(C)** were collected and prepared for primary culture. The collected tissues **(B**i**,C**i**)** were washed and dissected into small pieces (explant) before seeding on gelatin-coated culture dishes **(B**ii**,C**ii**)**. The explants were cultured in complete fibroblast (FB) medium and advanced DMEM (AD) medium **(B**iii,iv**,C**iii,iv**)**. Fibroblast outgrowth was found in both FB **(B**iii**,C**iii**)** and AD medium **(B**iv left**,C**iv left**)** while keratinocyte outgrowth was observed only in AD medium **(B**iv right**,C**iv right**)**. **(B**v**,C**v**)** Subculture at passage 1 (P.1) after primary culture reaching 90% confluency. **(D)** Tissues from excision at apex of ear pinna from fishing cats.

To preserve more genetic variations of fishing cats, cryopreservation of cell samples collected from natural resources is required. Under the permission of the National Park of Thailand (in the Animal Ethics section), six fishing cats from nature were obtained to collect samples, including a skin biopsy. Due to the nature of fishing cats capturing prey around wetland areas, we collected small skin tissues from the ear pinna, but not from other parts of the abdomen and leg area to avoid infection, of anesthetized fishing cats (see procedure of tissue collection in Figure 2A). The excision areas of the ear pinna were varied including deep and shallow excision of medial border of the helix and apex of the pinna (Figure 2A). The collected skin tissue (Figures 2Bi,Ci,Di–iii) was kept cold (4°C) in complete fibroblast medium (except a sample of male#2 (Figure 2Di) in normal saline solution) during transport to the tissue culture facility. Nevertheless, the transportation time was varied due to distances from sampling locations to the cell culture facility, from within a few hours to days (Table 1). The tissue explants of female#1 and male#1 (Figures 2Bi,Ci, Table 1) were cultured within 5 h after skin biopsy while the tissue from others took 1–3 days to reach the facility (Table 1). The primary culture of freshly collected pinna tissue from the ears showed that fibroblast outgrowth could be observed in 3 days from most explant tissues (Figures 2Biii,iv,Ciii,iv). However, epithelial-like keratinocytes [the morphology-based observation according to (42)] also expanded in advanced DMEM medium (AD) (Figures 2Biv right,Civ right), but not in fibroblast medium (FB) (Figures 2Biv left,Civ left), which reduced the proportion of keratinocytes/fibroblasts after passaging in DMEM-based medium (Figures 2Bv,Cv). The tissue explants of living fishing cats (male#1 and female#1) can be re-explanted at least three times and produced fibroblast culture sufficient to freeze for 30 cryovials (~100,000 cells/vial), Table 1. The ear tissues from male#2–3 and female#2–3 (Figure 2Di–iii, Table 1), with longer transportation time and tissue collection from apex of the pinna, did not give rise to any fibroblast derivation. It should be noted that tissue cell cultures collected from natural resources resulted in repeated fungal contamination, even in the presence of Amphotericin B. We also compared the duration of the medium change and found that the primary culture with daily medium changes produced more fibroblasts, while the medium change every 2/3 days caused 100% contamination (Figure 2).

### Primary culture of tissues from postmortem fishing cats

**Figure 3:**
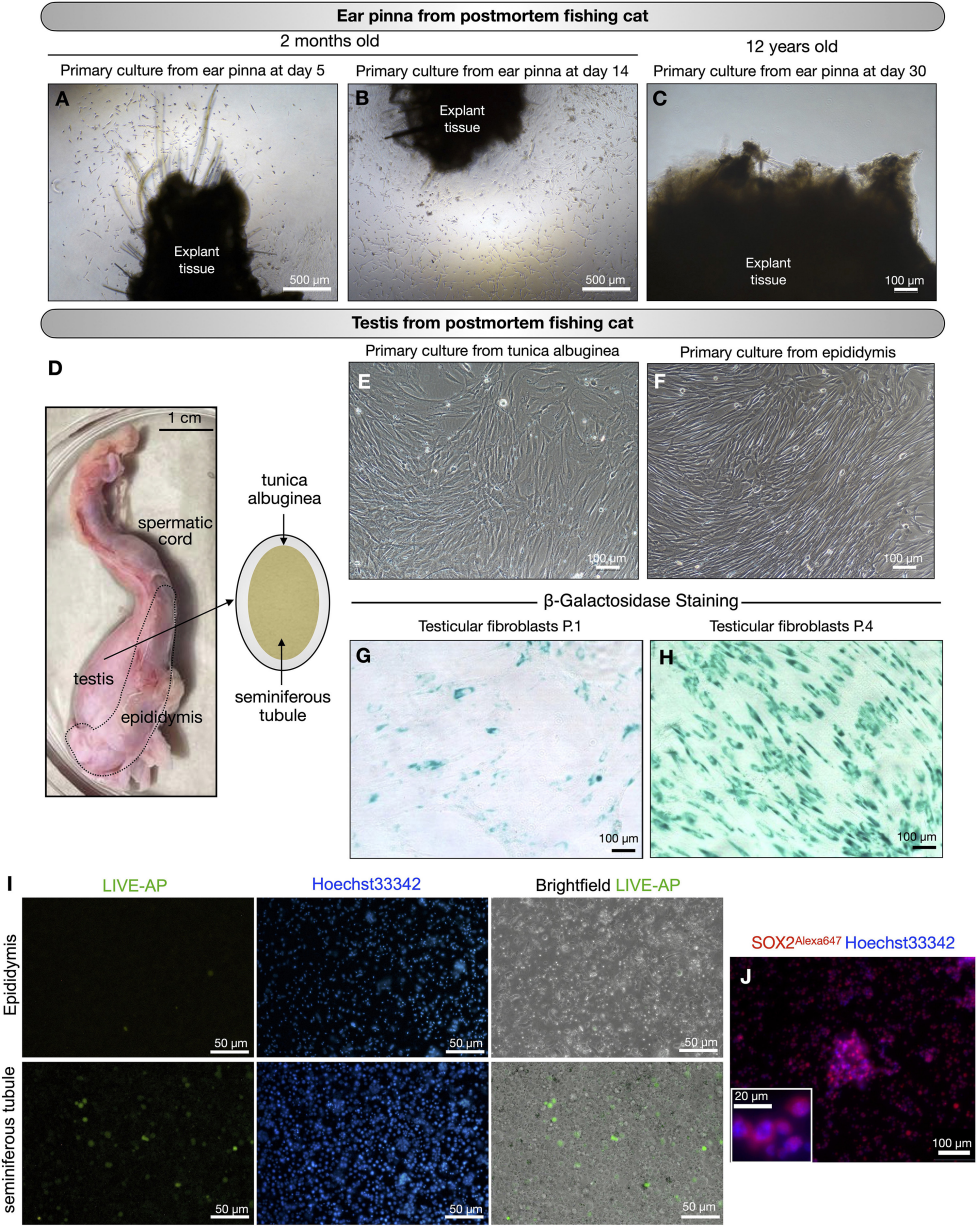
Primary culture of postmortem fishing cats. **(A–C)** Primary culture of tissues collected from ear pinna of postmortem fishing cats. **(A,B)** young fishing cat at age 2 months. **(C)** Adult fishing cat at age 12 years. No cells expanded from ear pinna explant in primary culture at day 30. **(D–J)** Primary culture of tissues collected from testis of postmortem fishing cat (adult at age 12 years). **(D)** Male reproductive organ of fishing cat collected for primary culture. **(E)** Fibroblast outgrowth from tunica albuginea explants. **(F)** Fibroblast outgrowth from epididymis explants. **(G,H)** Fibroblasts from the testes at passage 1 and passage 4 were tested for cell senescence. **(I)** Live alkaline phosphatase (Live-AP) staining was used to detect the presence of putative spermatogonial stem cells (SSCs) from seminiferous tubules and epididymis. Green color indicates possible signal for AP activity. **(J)** Immunostaining of seminiferous tubule extract to detect SOX2 protein (Red, Alexa647) with nuclear staining Hoechst33342. Inset shows zoom-up of SOX2-positive cells.

From postmortems of fishing cats at 2 months (female#7, Table 1) and 12 years (male#5, Table 1), we can retrieve two parts of the body, including the pinna of the ears (both female#7 and male#5) and the testes–epididymis (male#5). The ear pinna transplants were seeded 1-day postmortem. We derived fibroblasts from the ear pinna only from young female#7 (Figures 3A,B), but we could not obtain any primary cells after prolonged culture for a month from a much older male adult (male#5) (Figure 3C). Using domestic cats as a model, we investigated the possibility of extracting primary cells from the testes. Domestic cat testicular fibroblasts can be obtained and expanded, as shown in Supplementary Figures 1A,B. We used this procedure on a fishing cat sample (Figure 3D) and discovered that fibroblasts can be extracted from both the tunica albuginea and the epididymis of male adult (male#5, Table 1, Figures 3E,F). When comparing testicular fibroblasts between P.1 and P.4, we observed a stronger senescence signal (more blue color in cells) in P.4 (Figures 3G,H). This indicates that the usability of adult testicular fibroblasts is limited to only early passages (less than P.4). In addition, we found cells positive for alkaline phosphatase activity within the seminiferous tubules, but not in the epididymis (Figure 3I). AP activity was also detected in SSCs from domestic cats (Supplementary Figures 1C–F). Using immunofluorescence with an antibody to detect the SOX2 protein, we also found SOX2-positive cells in the culture of cells from the seminiferous tubules, indicating the plausible presence of SSCs (Figure 3J). Thus, from the postmortem fishing cat, we can cryopreserve cells from various sources, including the tunica albuginea, epididymis, and putative SSCs of seminiferous tubules.

### Delivery of DNA vectors to hard-to-transfect adult fishing cat cells by nucleofection

**Figure 4:**
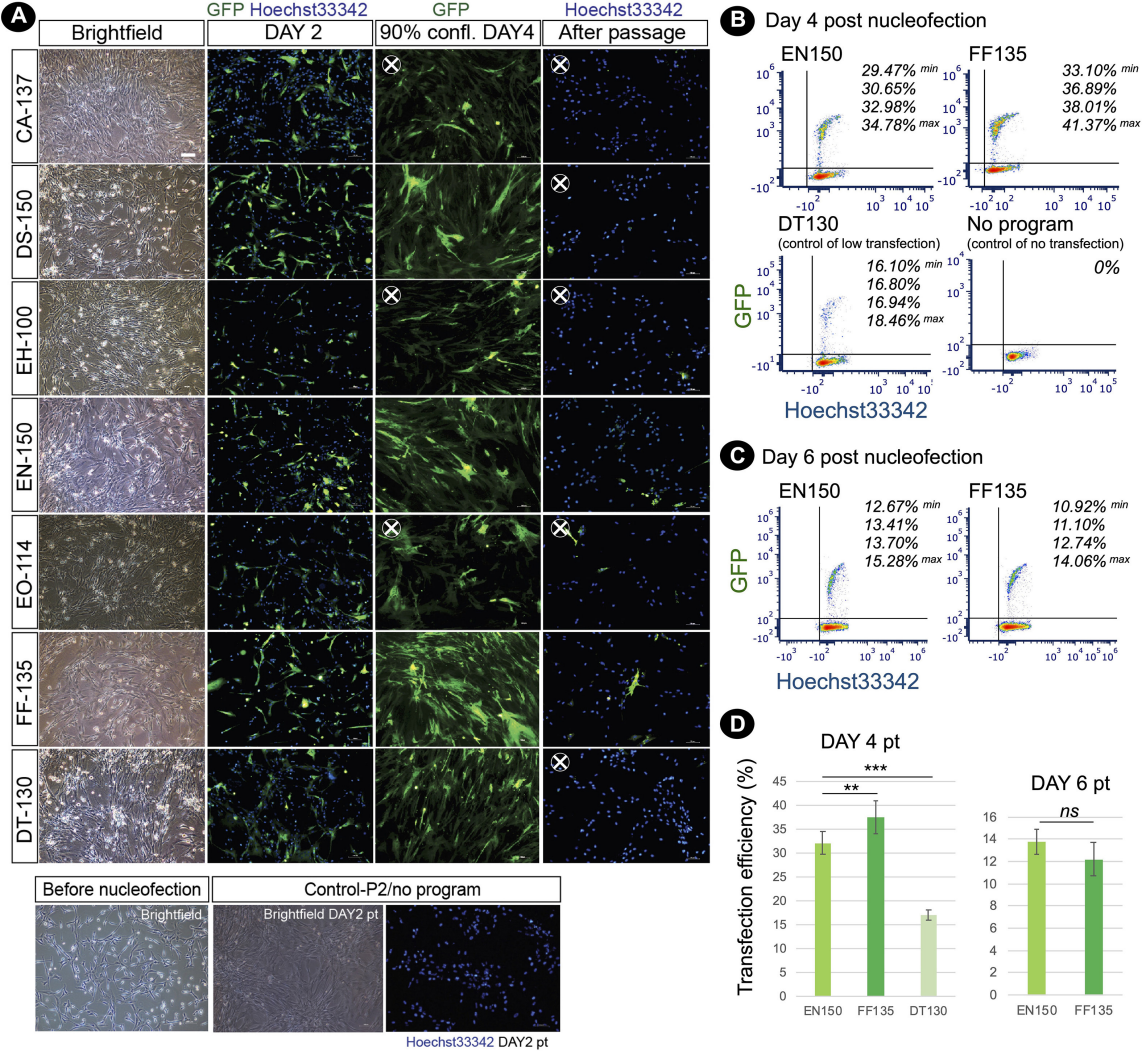
Nucleofection-based DNA delivery into adult dermal fibroblasts from fishing cat. **(A)** Fluorescence photographs show the fibroblasts transfected with pmaxGFP expressing GFP using the nucleofection method. Seven different nucleofection programs (CA-137, DS-150, EH-100, EN-150, EO-114, FF-135, and DT-130) were examined. Photos were taken on day 2, day 4 (90% confluency), and day 6 (day 2 after subculture) post-nucleofection. Nuclei were counterstained with Hoechst33342 (blue). At day 4 post-nucleofection, CA-137, EN-150, and EO-114 conditions contained a smaller amount of GFP+ cells and were removed for further analysis (X). After passaging, DS-150 and DT-130 conditions show a smaller number of GFP+ cells and were removed for further analysis (X). **(B)** Cells transfected with the best two nucleofection programs: EN-150 and FF-135 were analyzed by flow cytometry to quantify transfection efficiency. The representative contour plots show the percentage of double GFP+ and Hoechst 33342+ cell subsets (4 replicates) at day 4 post-transfection. DT-130 program was used as a control of low transfection, and “No program” indicates a condition of no nucleofection in the presence of P2 solution and pmaxGFP DNA. **(C)** as in **(B)**, the contour plots show the percentage of double GFP+ and Hoechst33342+ cell subsets (4 replicates) at day 6 post-transfection or day 2 after subculture. **(D)** Graph shows transfection efficiency of nucleofection (mean ± standard deviation) at days 4 and 6 post-transfection calculated from percentage of double GFP+ and Hoechst 33342+ cell subsets [*n* = 4, as shown in **(B)**]. Asterisks (** and ***) indicate significant differences (*p* < 0.05 and *p* < 0.01, respectively, Student's *t*-test), and “ns” indicates not significant (*p* < 0.05). pt, post-transfection; confl, confluency.

To enhance the limited proliferative capacity of fibroblasts, our objective was to optimize DNA delivery into fishing cat dermal fibroblasts as a further application for the next step of reprogramming. Nucleofection, performed using the Nucleofector^TM^ (Lonza) device, has been used for non-viral transfection of genetic materials with high efficiency into primary cells and hard-to-transfect cells. In this study, we performed DNA delivery using the 4D-Nucleofector^TM^ system. In general, each cell line requires a specific nucleofector solution (manufacturer's non-disclosed recipe) and an electric pulse program to transfer DNA to the cytoplasm and even the nucleus of cells. Here, our objective was to define the optimal Nucleofection^TM^ condition by testing seven programs specific for various mammalian cell lines, including human cells with the recommended Primary Cell 2 (P2) solution specific for mammalian dermal fibroblasts. Based on the expression of GFP, all programs could transfect fishing cat abdominal fibroblast cells. Each program provided different cell viability and transfection efficiency. On day 4 after nucleofection, when cells reached 90% confluency, differences in GFP expression became obvious, since three programs including DS-150, EN-150, and FF-135 showed outstanding performance. However, after subculture, cells transfected with EN-150 and FF-135 conditions still contained GFP expression (Figure 4). We used flow cytometry to confirm transfection efficiency and showed that the FF135 program had the best transfection efficiency with 33.10–41.37% GFP+ cells on day 4 after transfection (Figure 4B). The percentage of GFP+ cells was reduced by more than half on day 6 when the cells reached more than 90% confluence (Figure 4C).

**Figure 5:**
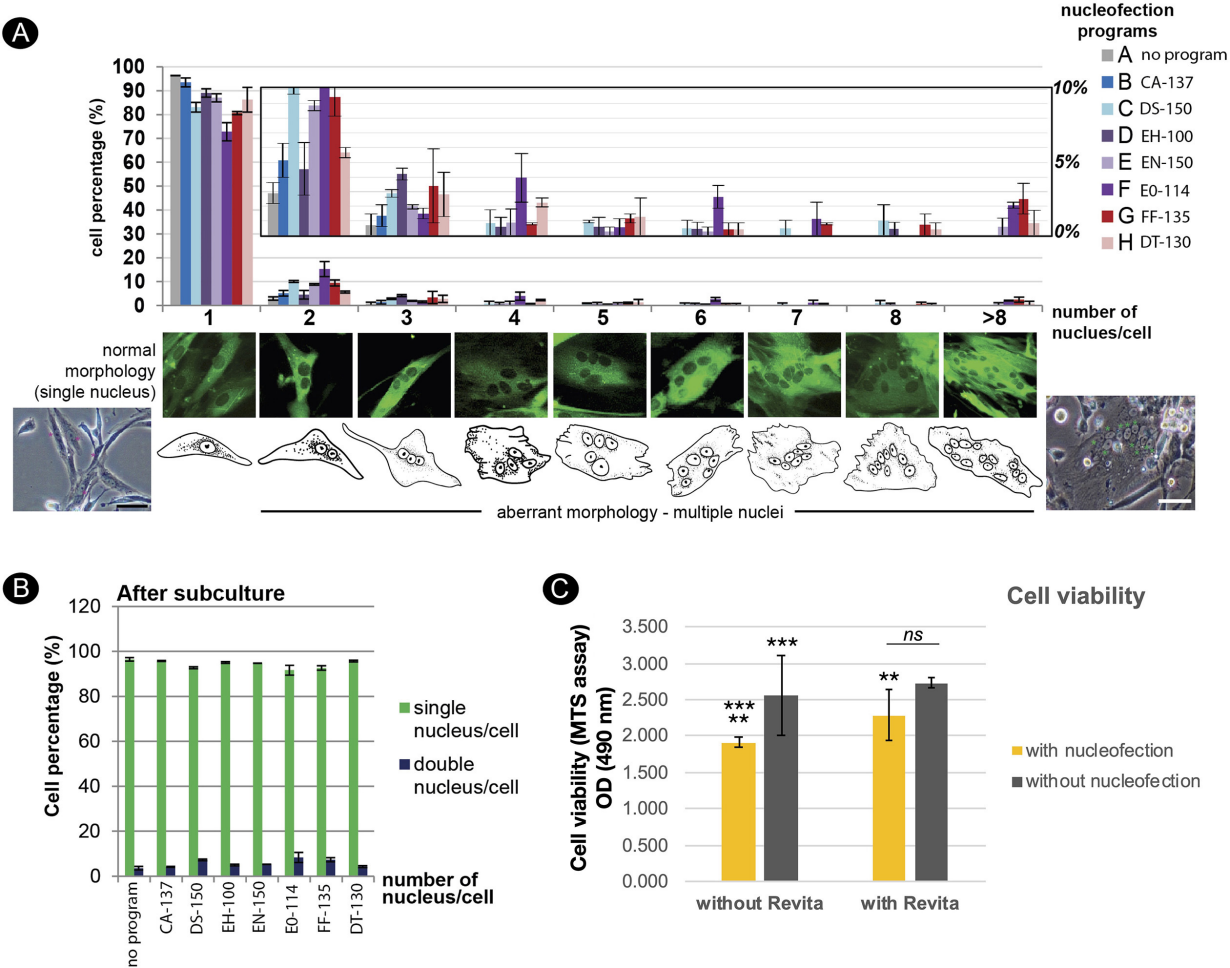
Effects of nucleofection on multinucleation and cell viability of adult dermal fibroblast derived from fishing cat. **(A)** Multinucleation occurred in different levels from various Nucleofector^TM^ programs. Top panel shows a graph depicting the incidence of multinucleation from different Nucleofector^TM^ programs. The Y-axis value represents the percent of transfected cells with one or more nuclei per cell (mean ± standard deviation). The X-axis indicates the number of nuclei per cell. The middle panel shows the morphology of GFP expressing cells with different types of multinucleation. The bottom panel shows brightfield images and cartoons of fibroblast cells with a single nucleus and multiple nuclei. **(B)** The graph indicates the number of nuclei per cell as in **(A)** from subculture of the transfected cells at day 3 post-passaging or day 7 post-transfection. Subculture of transfected cells removed multinucleation from all examined programs. **(C)** Graph shows cell viability of fibroblast cells after nucleofection with program FF-135 with or without RevitaCell^TM^ supplement. Cell viability was evaluated by MTS assay and quantified with spectrophotometer at 490 nm as shown in Y-axis. Mean indicates the average of absorbance values from four independent experiments with three technical replicates each. Error bar indicates standard deviation. Asterisks (** and ***) indicate significant difference (*p* < 0.05 and *p* < 0.01, respectively, Student's *t*-test), and “ns” indicates not significant (*p* < 0.05).

After nucleofection, some abnormal characteristics of the fibroblasts could be detected. Different degrees of multinucleated cells appeared in the nucleofected culture within 24 h. Among the two best Nucleofector^TM^ programs (EN-150 and FF-135) described above, FF-135 exhibited a higher degree of multinucleation (Figure 5A). However, this multinucleation could be eliminated by a one-time subculture of the condition transfected with FF-135 (Figure 5B). Furthermore, to improve cell survival after nucleofection, we treated transfected cells with RevitaCell^TM^ supplement (RC), which contains a ROCK inhibitor comparable to Y-27632 and Thiazovivin, and found that the addition of 0.5x and 1.0x RC improved survived cells in a similar way, which was twice higher than non-treated transfected cells (Figure 5C).

### Challenges to obtaining induced pluripotent stem cells from fishing cats for future conservation

**Figure 6:**
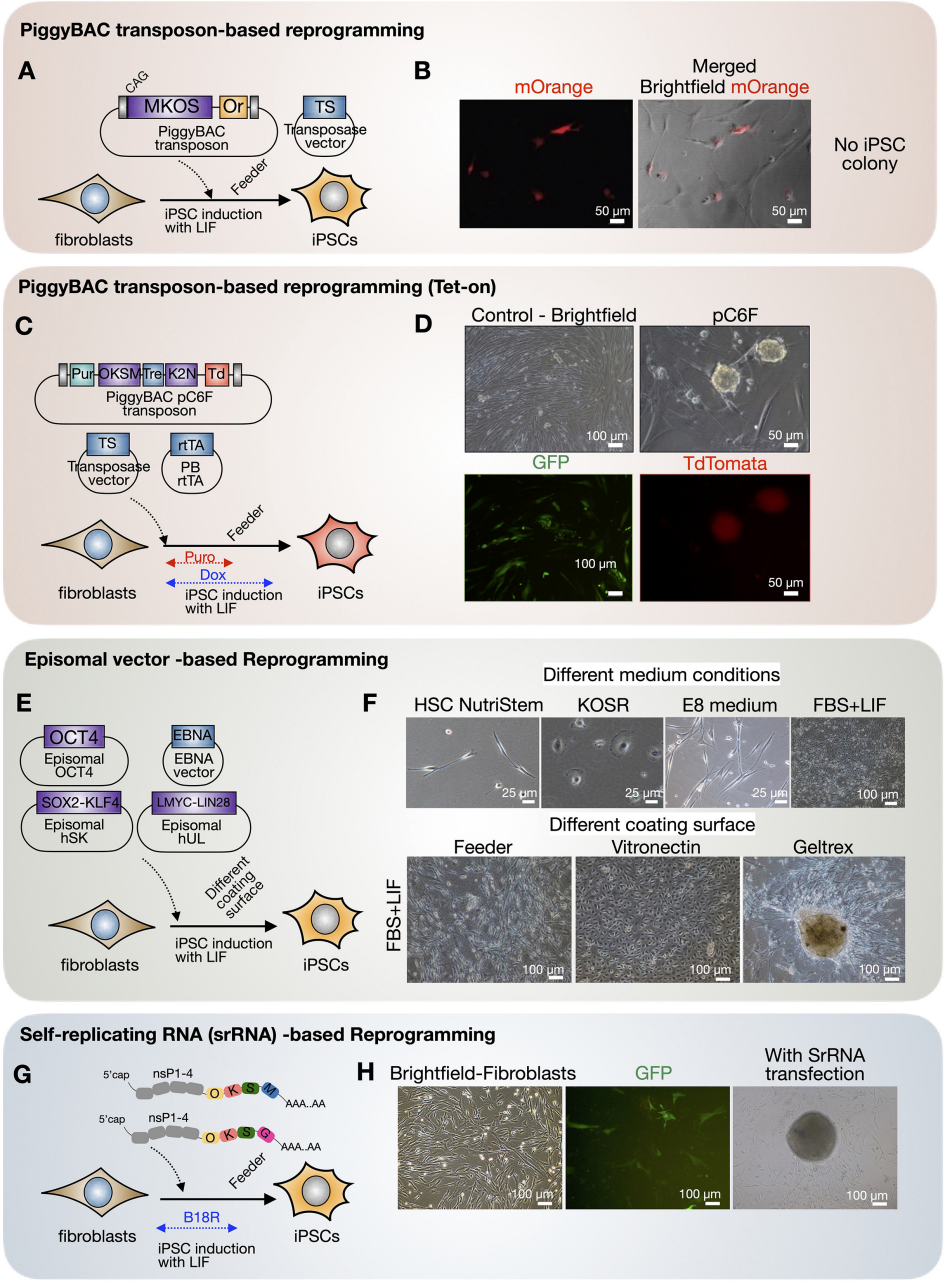
Cellular reprogramming of fishing cat cells *via* different virus-free approaches. **(A,B)** Fishing cat fibroblasts were reprogrammed with PiggyBAC transposon carrying mouse C-Myc, Klf4, Oct4, and Sox2 (MKOS) and mOrange (Or) as a reporter, together with an expression vector expressing transposase (TS). The fibroblasts expressed mOrange, indicating the successful integration of PiggyBAC transposon. **(C,D)** The fibroblasts were reprogrammed with PiggyBAC transposon carrying inducible reprogramming cassette composed of human OCT4, KLF4, SOX2, C-MYC (OKSM), and human KLF2 and NANOG followed by Ires-tdTomato. The transfection included transposase vector and PiggyBAC expressing rtTA. The transfected cells were selected with puromycin, and the expression of reprogramming factors was induced *via* Tet-On using doxycycline. The iPSC-like colonies appeared with tdTomato expression. **(E,F)** Reprogramming of fibroblasts using episomal vectors. The transfected cells were tested in various media including commercial HSC NutriStem and E8 medium, KOSR containing medium (KOSR), and media supplemented with 10% fetal bovine serum (FBS) and LIF. The induced cells were also replated onto different matrices including mouse-irradiated feeder, vitronectin, and Geltrex. **(G,H)** RNA-based reprogramming using self-replicating RNA (srRNA) expressing reprogramming factors. The testicular fibroblasts were transfected with srRNA and cultured in the presence of B18R protein before replating onto feeder cells. The iPSC-like colonies appeared 3 weeks after transfection.

Reprogramming of fishing cat fibroblasts was aimed to enhance cell propagation capacity *via* the induction of pluripotent state. First, we induced fishing cat fibroblasts with the PiggyBAC transposon–transposase system (mouse Oct4, Sox2, Klf4, C-Myc) by applying the nucleofection strategy described above with mouse reprogramming factors and mOrange as a reporter (Figure 6A). We confirm the presence of cells transfected with mOrange expression (Figure 6B) although iPSC colonies cannot be formed with this approach. In the second PiggyBAC transposon-based reprogramming strategy in fishing cat testicular fibroblasts, we used the Tet-On expression system of reprogramming factors and cell selection using puromycin (Figure 6C). The expression of tdTomato allowed us to monitor the expression of reprogramming factors (human OCT4, SOX2, KLF4, C-MYC, KLF2, and NANOG). We found that with this approach, iPSC-like colonies with tdTomato expression emerged (Figure 6D). However, colonies expressing tdTomato were not expandable in iPSC medium with human LIF. We also applied episomal-based reprogramming using human reprogramming factors (human OCT4, SOX2, KLF4, LMYC, LIN28) (Figure 6E). Cells transfected with episomal vectors were treated with various conditions (Figure 6E). With this approach, fishing cat cells could expand most in FBS-based medium and with Geltrex (Figure 6F). The fibroblastic characteristics were lost under Geltrex conditions, adopting more epithelial characteristics (Figure 6F), but not in mouse-based feeder cells (Figure 6F). After prolonged culture until the 3rd week of reprogramming, colonies similar to iPSC appeared in Geltrex (Figure 6F) but retained flat epithelial cells similar to those in vitronectin (Figure 6F). However, the selected colonies from Geltrex could not be propagated. Lastly, we tested whether RNA-based reprogramming can induce fishing cat cells based on Yoshioka et al. (38) and Yoshioka and Dowdy (39). We used self-replicating RNA expressing the OCT4, SOX2, KLF4, C-MYC, and GLIS1 proteins to induce the formation of iPSC colonies, but the same problems occurred from unexpandable clones (Figures 6G,H).

Thus, inducing and capturing pluripotency of fishing cats remain a major challenge. However, with these reprogramming approaches and an adjusted culture strategy, fishing cat fibroblasts can be reprogrammed to expandable intermediate cells, with episomal vector-based reprogramming (Figure 6), beyond the limitation of fibroblast passaging that we can keep in cryopreservation for future reprogramming success.

## Discussion

**Figure 7:**
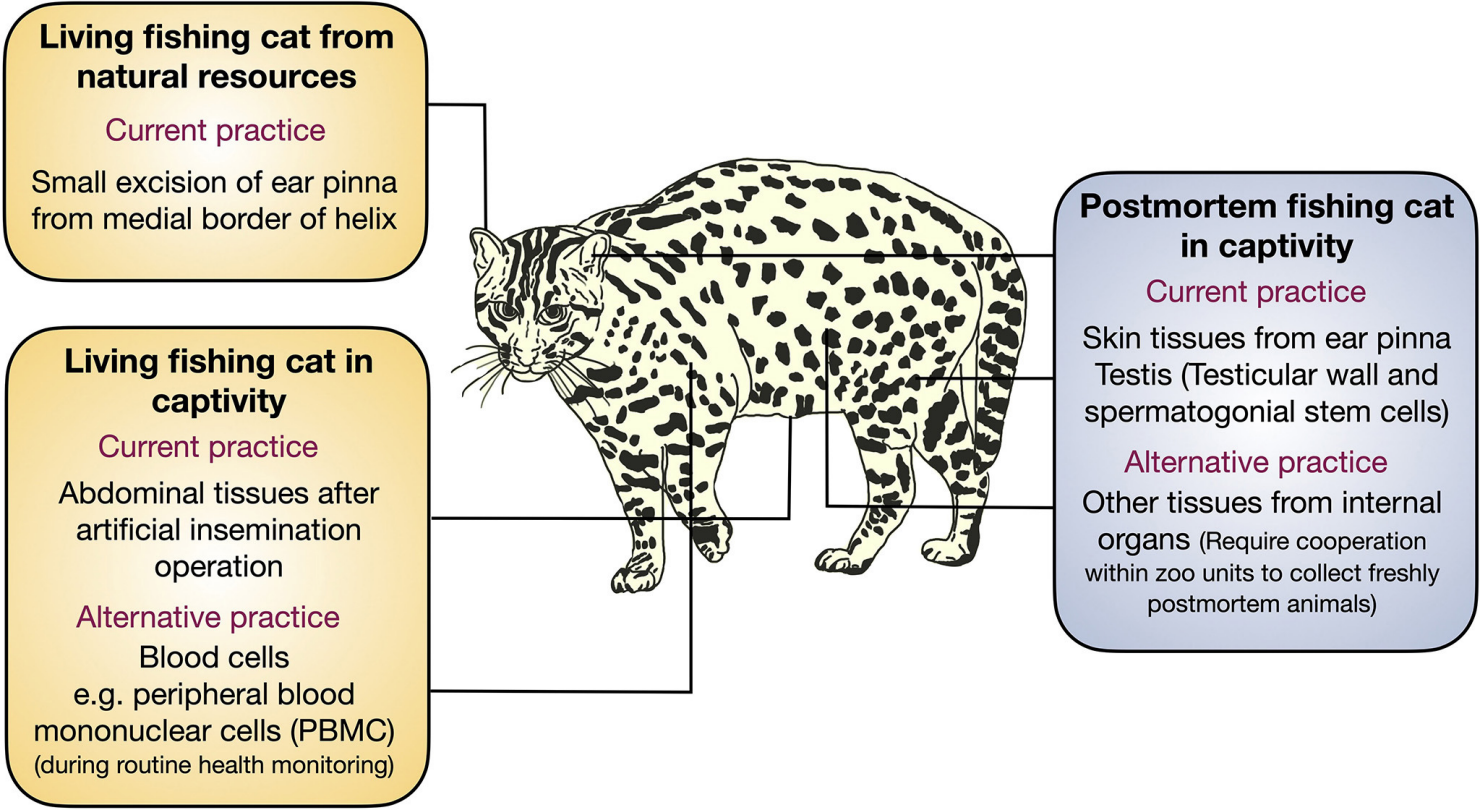
Tissue collection from fishing cat for conservation. Schematic illustration showing various sources of tissue collection for primary cell culture for cryopreservation in this study and suggesting the alternative sources for primary cells from the fishing cats.

Fishing cat cell biobanking is currently required for preserving its genetic makeup. Herein, we report the progress of our cryopreservation of fishing cats from several sources (summarized in Table 1) and provide examples and challenges of using adult somatic cells for cellular reprogramming. Embryonic or fetal fibroblasts are common sources for downstream applications, as embryonic fibroblasts exhibit a better regeneration process and wound/ligament healing than adult fibroblasts (40, 41). However, it is difficult to obtain sources of embryos from wildlife. Thus, adult cells are the only option. In a recent study, four cell lines were isolated from human skin tissue including keratinocytes, melanocytes, fibroblasts, and dermal microvascular endothelial cells, using tissue digestion method (42). However, due to limited sample collection in fishing cats and different cell compositions in skin tissues from the ear pinna, abdominal tissue, and even testes, we only used the explant tissue seeding method as demonstrated in domestic cats (31) for all samples, allowing us to compare the outcome of primary cell derivation. In this study, the adult cells can be derived from tissues from both living and postmortem fishing cats, but various sources contributed differently, summarized in Figure 7. From the living source, we can obtain fishing cat cells from the abdomen during AI and ear pinna, in particular medial border of helix, from ear clipping. The small pieces of the ear pinna from living fishing cats provided a higher number of fibroblasts than the postmortem ones for cryopreservation, whereas the collection of testes from the postmortem fishing cat provided sufficient cryopreserved sources of cells, including fibroblasts of the tunica albuginea and epididymis, and mixed cell populations containing putative SSCs from seminiferous tubules. Therefore, we recommend collecting testis for cell culture from freshly dead wild animals will provide a good source for biobanking. Although it has been demonstrated in other mammals that fibroblasts can be produced from the ear tissues stored for several days (43, 44) or even months (45), there were a number of factors that contributed to the failure of this experiment in the postmortem fishing cat. With strict protocols (complete fibroblast medium for tissue collection and transportation and storage at 4°C before tissue culture), the possible factors might be age (12 years old) and the long duration of animal death left at a high temperature, which could affect the prolonged lack of blood supply to the dermal tissues in the ears as well as decreased humidity, likely leaving tissue more desiccated and with a smaller number of viable expandable fibroblasts. This scenario appears to be similar to the failure to isolate fibroblasts from elephant carcasses (more than 20 h after death), as demonstrated by Siengdee et al. (23). In contrast, the successful primary culture from the same animal's testes could be attributed to the tissues being kept in a more favorable and humid environment within the body prior to tissue collection.

Although it was not unexpected that keratinocytes appeared from ear pinna explant culture that contained intact epidermis, keratinocytes can be eliminated after continuing culture in DMEM high-glucose medium with FBS, as appeared in Vangipuram et al. (46), for human fibroblast derivation and Siengdee et al. (23), for Asian elephant fibroblast derivation. In addition to medium types, cell culture and supplementation procedures also affected the culture (23, 42, 46). Whether prolonged culture or more passaging of fibroblasts from fishing cats leads to cell senescence, the fibroblast is no longer usable for other applications. Obstacles can be overcome by adding bFGF, a typical cytokine known for its ability to support human fibroblast cell proliferation in a dose-dependent manner through the ERK1/2 and JNK pathways (47), which improved cell proliferation in our culture. Therefore, the frozen stock of early fibroblasts should be made of as many as possible, which is also limited to the amount of tissue harvested.

In addition, as it is routine that after fishing cats or other male wild animals in the zoo are dead, the veterinarian will collect testes to extract viable sperms for cryopreservation. This opens the possibility of spermatogonial stem cells (SSCs)–adult stem cell culture [reviewed in Sahare and Suyatno (48)] or even further reprogramming to obtain more potential stem cells from testes of postmortem fishing cats. In the literature, the existence of SSCs can be detected by the presence of alkaline phosphatase (AP) activity (49, 50) and the detection of SOX2, which is present in possible SSCs [SOX2+ adult stem cells mentioned in Arnold et al. (51)] and in porcine testis (50, 52). This population of SOX2+ cells, a type of adult stem cell in mice, can repopulate the testes with ablated spermatogenesis and restore spermatogenesis (51). In this study, we also show that the presence of living SOX2 + cells and AP+ cells can be obtained from a postmortem fishing cat. Thus, the finding of viable SSCs in dead fishing cats provides additional sources for cryopreservation and could pave more ways to preserve more wild animals immediately postmortem in captivity.

Delivering genetic material to adult fishing cat cells (e.g., for reprogramming) is another challenge due to the nature of the hard-to-transfect cell type with a low division rate compared to embryonic sources. Nucleofection, an electroporation-based transfection method that enables direct DNA delivery into the nucleus, can solve the problem as we showed that the diverse range of transfection efficiency of different nucleofector programs could indeed deliver DNA to the fishing cat cells. Similarly, this technique is one of the most suitable non-viral transfection methods to deliver DNA to dermal fibroblasts in various species, including humans, rats, and mice (33, 35, 36, 53–55).

As fibroblasts have limited capacity to expand, preserved cells for better usability are through reprogramming of fibroblasts to iPSCs, which have high cell potency for self-renewal and differentiate to all types (56, 57). Felid reprogramming has already been performed in some wild and domestic cats with retrovirus/lentivirus-based approaches (29, 30, 58). Since then, there has been no success with the non-integration approach of wild felid reprogramming. In this study, we examined a mouse/human reprogramming approach to induce fishing cat cells without using a virus to deliver reprogramming factors. iPSC colonies appeared from episomal vectors with human OCT4, SOX2, LMYC, KLF4, and LIN28 (59) and PiggyBAC transposon with human OCT4, SOX2, KLF4, C-MYC, KLF2, and NANOG (37). However, putative iPSC clones cannot be expanded under mouse iPSC (LIF) or human iPSC maintenance (bFGF) conditions. The nature of adult fibroblasts with early senescence or short replicative lifespan [possibly from various factors, such as telomere length, epigenetic modification, and mitochondrial function, as reviewed by Strässler et al. (60)] is one of the plausible reasons for partial reprogramming from fishing cat adult cells. Reprogramming efficiency (number and time to the first appearance of an iPSC colony) from adult cells may be affected by donor age, as demonstrated in mice (61). Although we began reprogramming from early passages of somatic cells, the cells divided slowly and required a longer period of culture before being sufficient to begin reprogramming, and this could affect the efficiency of iPSC induction, as shown in Trokovic et al. (62). Furthermore, we found that conditions with more reprogramming factors resulted in better iPSC colony formation, which is consistent with the finding of Lapasset et al. (63), that adding Nanog and Lin28 to Yamanaka factors supports more iPSC induction from senescent human fibroblasts. Therefore, maintaining pluripotent states or inducing the fully reprogrammed state of the fishing cat remains difficult. However, reprogramming to a partial state with these reprogramming factors increased the expandable capacity of the fishing cat cells, possibly due to rejuvenating cells and contributing more cryopreserved cells for biobanking.

Taken together, biobanking strategies for preserving the genetics of fishing cats are constrained by the availability of samples from nature and captivity, fibroblast potency, the delivery of genetic material to difficult-to-transfect cells, and the achievement of a fully reprogrammed state. However, we were successful in preserving somatic cells from living and postmortem fishing cats for future conservation technologies to prevent fishing cat extinction.

## Data availability statement

The raw data supporting the conclusions of this article will be made available by the authors, without undue reservation.

## Ethics statement

The animal study was reviewed and approved by Zoological Park Organization of Thailand under the Royal Patronage of H.M. the King (Protocol number: 630960000030 granted to PI of the project AT). Permission to work with wild fishing cats in the Natural Parks of Thailand was granted by Department of National Park Wildlife and Plant Conservation-Thailand (Permission number: Tor Sor (in Thai) 0907.4/17939 (Issued date: 22/09/2021).

## Author contributions

WSu and AT designed experiments and wrote the manuscript. SK and NS conducted primary culture. RP and IH derived testicular fibroblasts and reprogrammed them with PiggyBAC transposons. RB and WSi performed nucleofection and flow cytometry. All authors performed cellular reprogramming assay, contributed to the article, and approved the submitted version.

## Funding

This research was supported by the Research Fund for DPST Graduate with First Placement (grant number 029/2558), the Development and Promotion of Science and Technology Talents Project (DPST), and the Institute for the Promotion of Teaching Science and Technology (IPST), Thailand, the Research Grant of Faculty of Science, Burapha University (Grant No. 2559/N2 to WSu), the Research Grant of Burapha University through National Research Council of Thailand (Grant No. 92/2560 to WSu), and the Research Grant of Zoological Park Organization under the Royal Patronage of H.M. the King through National Research Council of Thailand (Grant No. 6309600000030 to AT).

## Conflict of interest

The authors declare that the research was conducted in the absence of any commercial or financial relationships that could be construed as a potential conflict of interest.

## Publisher's note

All claims expressed in this article are solely those of the authors and do not necessarily represent those of their affiliated organizations, or those of the publisher, the editors and the reviewers. Any product that may be evaluated in this article, or claim that may be made by its manufacturer, is not guaranteed or endorsed by the publisher.

## Supplementary material

Supplementary Figure 1 Primary culture of cells from testis of domestic cat (*Felis catus*). **(A)** Fibroblast outgrowth from explants of tunica albuginea. **(B)** Fibroblasts before cryopreservation. In **(A,B)**, cells were cultured in complete fibroblast medium. **(C–F)** Cells from seminiferous tubules of testis were extracted and stained with live alkaline phosphatase (Live-AP) staining (green color indicates possible signal for AP activity).
