# Supplementary figures and images for: Establishment of fishing cat cell biobanking for sustainable conservation
Source: Front Vet Sci. 2022 Nov 10;9:989670. doi: 10.3389/fvets.2022.989670 (PMC9684188; doi:10.3389/fvets.2022.989670)

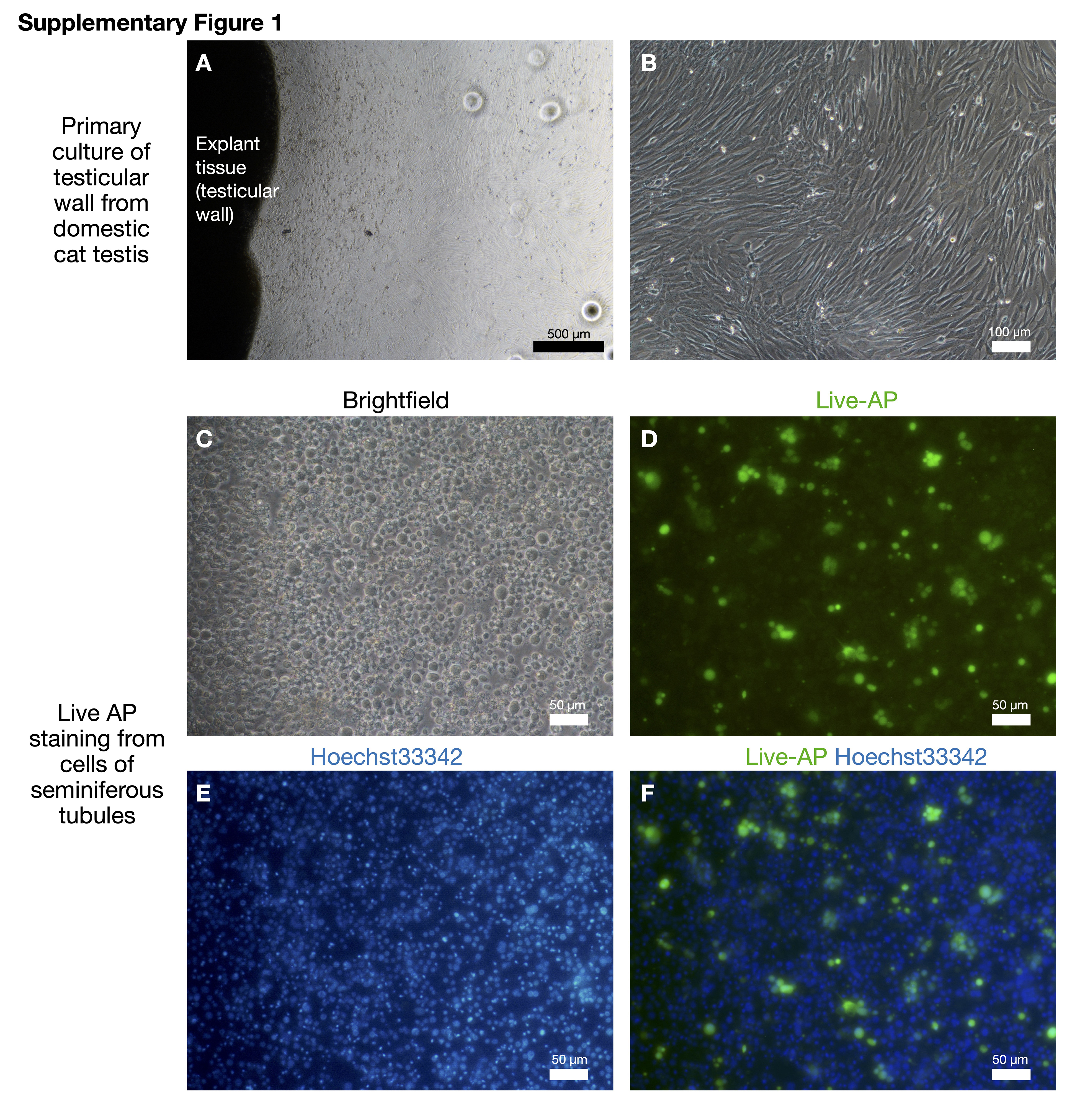

Supplement: Supplementary Figure 1 — Primary culture of cells from testis of domestic cat (Felis catus). (A) Fibroblast outgrowth from explants of tunica albuginea. (B) Fibroblasts before cryopreservation. In (A,B), cells were cultured in complete fibroblast medium. (C–F) Cells from seminiferous tubules of testis were extracted and stained with live alkaline phosphatase (Live-AP) staining (green color indicates possible signal for AP activity). [file Image_1.JPEG]
